# The central role of mindful parenting in child’s emotional regulation and human flourishing: a blueprint perspective

**DOI:** 10.3389/fpsyg.2024.1420588

**Published:** 2024-06-26

**Authors:** Antonella Sansone

**Affiliations:** Faculty of Society and Design, School of Psychology, Bond University, Robina, Gold Coast, QLD, Australia

**Keywords:** mindful parenting, child’s emotional regulation, human flourishing, blueprint, attachment, mindfulness practice, maternal mental health, supportive community

## Abstract

This article provides an innovative perspective of emotional-regulation and human flourishing which acknowledges the fundamental role of early parent–child experiences in shaping brain structure and functioning involved in emotional regulation and the central role of mindful parenting in facilitating emotional regulation in both parent and child (co-regulation). In this perspective paper the author underlines not only the central role of emotions and emotional regulation in human development and flourishing, but also the importance of maternal mental health, mindfulness, and a connected supportive community during pregnancy and postnatally in facilitating emotional regulation in both the caregiver and the infant and thus promoting secure attachment. The role of alloparenting and how we evolved to share childrearing is introduced, and emotional regulation is described not as an individual phenomenon but a relational embodied process. The associations between right brain functioning, mindfulness and secure attachment, all leading to emotional regulation, wellbeing, and resilience are described. Sharing findings and perspectives offer an opportunity for insights and reflection upon what strategies could be created to promote relational emotional regulation and wellbeing in early life, thus human flourishing leading to a peaceful society.

## Introduction

For centuries in Western culture emotions have been undervalued and regarded in a controversial way and as enemies of rationality and disruptive of cooperative social relationships. However, when well-regulated and well developed, they may be our finest form of rationality (MacMurray, 1992).

Emotional systems play a central role in human brain and dynamically interact with more evolved cognitive structures. Emotions change sensory, perceptual, and cognitive processing and guide behavior. Narvaez (2014) posits that moral development derives from earliest socio-emotional experiences with our caregivers. Therefore, emotions guide our perception of the world, our memories of the past and even our moral judgment of right and wrong, enabling us to respond to the current situation effectively and adapt. Yet, psychological theories often consider emotion and cognition as separate entities. Components of emotion systems (arousal, action tendencies, prospective motor control, intention) are generally placed in the brain stem, hypothalamic structures, and cerebellum. The brain stem develops during embryonic stage and the networks are integrating across different areas of the brain (Panksepp, 1998). Affective feeling links brain stem, paralimbic and prefrontal structures.

Emotions and cognition often overlap throughout the brain. In fact, many of the brain systems are involved in both domains (Panksepp, 1998). In the neuronal cortex there is no distinction between cognition and emotion. Emotion and cognition work as a functional unity and are linked to behavior (Lewis, 2005). There is no emotion without thought, and thoughts in general evoke emotions. Greenspan and Shanker (2004) propose that emotions arise from multiple factors, including physical experiences, signals from others and meaning making. These components later become the source of a child’s secure attachment, linguistic and cognitive advancement, and reflective capabilities. Caregivers’ attuned responses to the infant’s body cues, which express emotions and needs, influence the infant’s subjective experience of sensations (including pain), affect and cognitions and her or his ability to cope with challenging emotions.

This paper perspective aligns with and empirically extends the current view in both developmental psychology and moral philosophy that emotions are infused with reason; that emotional regulation is primarily developed through early relationship with our primary caregivers; and that emotional regulation is a central element of moral virtue development and related human flourishing. Ancient philosophers like Aristotle had an integrative view of morality beyond reasoning (Aristotle, 1988). He argued that emotions had to be well trained to lead to human and moral virtues. All virtues fit into a larger worldview of human flourishing (eudaimonia) and excellence (arête). My perspective takes up a similar broader view of morality that has an emphasis on flourishing in terms of not only psychology but also neurobiology. Moral virtues development emerges from physiological emotional co-regulation between a child and caregiver in early life. It includes the acknowledgment of the role of the mammalian caregiving nature and its interactive nature (Sansone, 2021).

Under evolved conditions, humans aim for social flourishing, which is the development of emotional co-regulation and cooperative social abilities that prepare one to live life with wisdom (Narvaez, 2014). Poor relational experiences during sensitive periods of development hinder flourishing. Narvaez holds that human flourishing emerges from experiences of caregiver’s receptivity to the child’s needs in the moment, habitual reciprocal experiences that build empathy, compassion, and sympathetic action. An ethics of love advances flourishing (Fromm, 1956). For those who experienced abuse or neglect the path toward emotional regulation, trust, empathy, and flourishing may be hard. If student’s flourishing is the aim of education (Kristjánsson, 2019), a neo-Aristotelian view, then mindful parenting, which enhances the cultivation of emotional regulation and relational embodied processes, can set the foundations of student flourishing. The blueprint perspective presented in this paper follows new directions in questioning how to educate young people toward a life of flourishing from life before birth.

Comprehending human flourishing requires an understanding of human development in the broad sense. Western longstanding assumptions that emotions are “nonrational, arbitrary, and subjective” have led to theories and practices that undermine humanity’s essence (Johnson, 1993, p. 132). Narvaez (2014) conceives human flourishing as corresponding to the fluidity of human development and interconnectedness and interdependence of the many systems it comprises. In line with this view, this paper embraces an appreciation of our original mammalian cooperative nature and powerful nature of social and emotion development. These systems develop interdependently within the interrelationships with all entities in Nature.

There is a lot of meaningful overlap between the position advanced in this paper and the literature on phronesis (practical wisdom), especially as concerns emotional regulation and its part in moral virtue development and a flourishing life (Kristjánsson and Fowers, 2023). The new science of virtue suggests that virtues are the habitual traits that make it possible to live a good or flourishing life, and phronesis is then considered to encompass the wisdom an individual adopts to choose which virtues are appropriate in a specific situation, so that action leads to that flourishing life (Kristjánsson and Fowers, 2023). Phronesis is central because flourishing is seen to include the harmonious implementation of virtues of character within a fulfilled life. Therefore, flourishing is a complex concept within the new science of virtue. There is a need to create a new model of virtue development and flourishing by integrating elements of neo-Aristotelianism and phronesis with various current empirical findings from developmental science, neuroscience, mindfulness, pre-and perinatal psychology, and education. Virtue development implies cultivating apt emotional responses to specific situations. Emotional regulation is seen as one of the functions of phronesis leading to virtue development. Virtue development is a topic in the intersection of developmental psychology, including pre and perinatal development, moral psychology, and moral philosophy.

Section one of this paper describes the relationship between synchronized interactions between parent and child and the origins of emotional regulation and moral virtues development, and the possible adverse effects that occur when this affect synchrony is hindered and not followed by regulation. Section two underlines not only the central role of emotions and emotional regulation in human development and flourishing, but also the importance of maternal mental health, mindfulness, and a connected supportive community during pregnancy and postnatally in facilitating emotion regulation in both the caregiver and the infant and thus promote secure attachment. In section three, I propose that a blueprint of co-regulation between mother and baby forms during pregnancy and grows particularly fast during infancy. In section four, I use evidence and my insights to highlight how mindfulness-based support helps parenting and child development through cultivation of emotion regulation, improved co-regulation, right-to-right brain communication and ought thus to be a public health priority. Finally, in section five, the paper concludes with clinical implications and future directions.

Sharing findings and perspectives offer an opportunity for insights and reflection upon what strategies could be created to promote relational emotional regulation and wellbeing in early life, thus human flourishing.

## Affect synchrony and the origins of emotional regulation

We know that the brain is a system of two brains, each of which has very different structural and functional properties, though complementary. The right brain has become of particular interest to the developmental sciences, as it undergoes a growth spurt and is dominant in the first two years from about the 25th gestational week, before the verbal left developing later (Schore, 2005). This growth is only partially led by the genome, being mostly shaped by the emotional communications between caregiver and infant within the early attachment relationship. Because the right hemisphere is dominant for the emotional and corporal self, the experience-dependent maturation of the right brain plays a very important role in early development of the self (Schore, 1994).

Models have moved from Piagetian theories of cognitive development to psychobiological models of social–emotional development. Psychological sciences and psychiatry are moving from cognition to emotion as the central force in development and psychotherapy. The emergence of affective neuroscience has reflected into a new focus on the role of the right hemisphere for processing affective states and of the limbic system, the brain system that receives subjective information about emotions that guide behavior and allow the individual to adapt to a rapidly changing environment.

A substantial body of research reveals that the right brain is also the locum of essential self-regulatory structures which development is facilitated by attachment relationships. The primary goal for the infant is the creation of emotional communications and the development of self-regulation (Schore, 2005). A large body of evidence indicates that the infant’s need for bonding and interactive emotional communication is present in prenatal life (Chamberlain, 2003; DiPietro, 2010; Sansone, 2021; Verny, 2021) and co-regulatory mechanisms can be facilitated at this critical time through the mother’s mindful awareness of her unborn infant as a sentient conscious being capable of relational engagement (Sansone, 2021, 2024).

The infant’s earliest interactions with the social environment occur through their developing motor and sensory capacities, especially hearing, smell, taste, and touch (Weller and Feldman, 2003; Ferrari et al., 2016). At around eight weeks, the social and emotional capacities progress dramatically. Through face-to-face interactions, the mother and infant engage in nonconscious facial, vocal, and gestural communications and synchronize their affective behavior. These emotional transactions expose the infant to high levels of social and cognitive information. The responsive mother picks up the nonverbal cues of her infant’s internal arousal and psychobiological states, regulate them, and communicates them back to the infant (Schore, 2021). This process of responsiveness consists of a coordination of engagement, disengagement, and re-engagement. The empathic attuned mother picks up the infant’s cues for re-engagement, thus synchronizing her interactions. The synchronized interactions promote the infant’s capacity for emotional regulation and are fundamental to her or his healthy affective development. The dyad creates mutual regulatory systems of arousal, or co-regulation.

The mother’s regulation of moments of misattunement or rupture in the mutual interactions allows for the infant’s development of self-regulation. To be able to regulate rupture and repair the caregiver needs be able to monitor and regulate her own (especially negative) affects. The stress experienced by the infant through a misattunement allows the infant to learn to regulate her or his negative affect. By re-experiencing positive affect following negative experience the child learns that negative affect can be tolerated, and that relational stress can be regulated. Infant resilience emerges from this smooth transition from positive to negative and back to positive affect and is an indicator of adaptive capacity, secure attachment, and optimal mental health (Schore, 2005). Therefore, attachment is the result of the regulation of biological synchronicity within and between organisms. The attuned mother minimizes the infant’s unpleasant states by comforting her or him while maximizing the positive affective states in interactive play. These predictable affective interactions with a primary caregiver provide the infant with a sense of safety and curiosity for the exploration of new socioemotional and physical environments. This ability is a marker of secure attachment, adaptive infant mental health, and human flourishing. These attuned interactions teach the child human virtues such as empathy, compassion, mutual understanding, and mutual respect, listening, trust and intimacy. Human beings’ moral development and capacity for ethics, therefore, arises from earliest socio-emotional experiences with our caregivers (Narvaez, 2014).

Through these interactive emotional transactions – coordinated visual eye-to-eye cues, auditory vocalizations, and tactile and body gestures expressing emotions - the child also learns how to communicate emotional states from the mother’s ability to communicate her own emotional states. These emotional communications between caregiver and infant correspond to an interpersonal neurobiology of right-to-right brain communications or neurobiology of attachment (Schore, 2021; Siegel, 2023). Although the left hemisphere is dominant for verbal language development, the early right hemisphere is more important to the broader aspects of communication, especially bodily, through all stages of life. Attuned responses to infant cues help the infant to organize his emotional and physiological experience leading to reflective functioning. Emotions and affect form “the source of symbols, the architect of intelligence, the integrator of processing capacities, and the psychological foundation of society” (Greenspan and Shanker, 2004, p. 46). So, emotional regulation can also form moral virtues, which in turn can be reinforcing tools for emotional regulation in adulthood.

When these positive predictable interactions and emotional transactions with the child do not occur, for example due to the parents’ low emotional awareness and difficulties in emotional regulation consequent to high levels of stress, depression, anxiety or psychiatric conditions, there are possible adverse effects on the child’s ability to regulate his or her emotions and other areas of development (Zimmer-Gembeck et al., 2022). I assume that the development of relational and communication skills and moral virtues may also be impaired.

The following section explores the role of communal caregiving and shared childcare in supporting maternal mental health, emotion co-regulation between caregiver and child, and more extensively, individual, and societal flourishing.

## Alloparenting, emotional regulation, mental health, and human flourishing

Although the mother is usually the primary attachment figure in the first year, the child in the second year forms another important attachment relationship to the father, allowing the child to have a different arousal regulating and affect-attuning experience. In indigenous and traditional societies, the multiple attachments a child is exposed to within a connected supportive community, promotes optimal emotional, social, and moral development and a peaceful community (Konner, 2005; Sansone, 2021). As a result of the different interactions with multiple caregivers and shared care, the infant forms internal working models of attachment, interactive representations of attachment relationships, which encode patterns of affect regulation and coping strategies for maintaining basic regulation and positive affect in the face of environmental challenges and stressors. Human capacities for empathy and mutual understanding evolutionary begun as a cooperative process through alloparenting or shared care (Hrdy, 2009). As humans, we have evolved to share childrearing and through community support, mother and child learnt to trust every member of the community and rely on their mindful care. To be able to trust them, mother and child had to be able to see the mind of the other – their feelings, thoughts, attention, awareness, and intention. This capacity for mind-reading and trust in receiving attentions from the community developed in the baby by default and became a source of self-regulation in both caregiver and infant (Sansone, 2021). Human mothers became naturally skilled at tuning into the internal states of the infant and facilitate emotional regulation. The right hemisphere that has been imprinted and organized by early relational experiences will remain for the rest of life dominant for the unconscious reception, expression, communication, and regulation of emotion, an essential function for creating and maintaining social relationships, especially intimate ones (Dimberg and Petterson, 2000). In a community in which these processes and an integrated communication between the caregiver and infant and between other community members and caregivers are supported, energy and information flow are shared with resonance. There is attunement with oneself as well as with the others - intra and interconnection (Siegel, 2023). The integrated communication between mother and infant during the first months facilitated by social resonance allows the two systems of the brain, left and right and all their functions involved, to integrate and foster a resilient life and human flourishing.

The right brain is also dominant for the regulation of fundamental physiologic, endocrinologic, immunologic, and cardiovascular functions, thus controlling vital functions that support survival and enables the organism to cope with stress. A growing body of data indicates a strong association between dysfunctions in mother-infant interactions, early programming of the hypothalamic–pituitary–adrenal axis, and brain development in pre-and perinatal critical periods and adult health and disease (Matthews, 2002; McGowan et al., 2008; Glover, 2014). Maternal stress, depression and anxiety during pregnancy and post-partum impact mother’s sensitivity and responsiveness to her infant, thus mother-infant interactions and attachment and increases the risk of emotional, cognitive, behavioral, and social problems in children (Mason et al., 2011; Hayes et al., 2013; Brassel et al., 2020).

The Polyvagal Theory (Porges, 2011) further contributes to our understanding of the physiologic effects of attuned emotional interactions between parent and child. This early affect synchrony helps establish optimal vagal tone, which equals with proper functioning of the visceral organs controlled by the vagal nerve, which in turn is connected with brain centers. Both emotion regulation and prosocial abilities are tied to vagus nerve functioning, which is fostered by responsive parenting in the first years of life (Porges et al., 1994; Calkins, 1997). The vagus nerve is the tenth cranial nerve and the primary nerve of the parasympathetic nervous system, which is implicated in the regulation of multiple biological systems. When it functions poorly, a variety of adverse health outcomes can take place (digestion problems, such as irritable bowel, neuronal communication, such as seizures, and mental health, such as depression) as well as inflammation, a backdrop for many diseases (Groves and Brown, 2005), especially in our modern world. When functioning well, the vagus nerve improves physiological self-regulation (e.g., of glucose), attention, and emotional and behavior regulation as well as interpersonal interactions (Porges et al., 1994; Kok and Fredrickson, 2010).

Therefore, the vagus nerve also influences emotion and emotion regulation (Calkins, 1997; Porter, 2003). Recall how the right brain hemisphere, involved in emotions and nonverbal interactions, develops rapidly in early life. The vagal system is lateralized in the right hemisphere and tied to emotional regulation abilities. Research found that children with higher vagal tone, an indicator of good vagal functioning, were more cooperative and giving (Eisenberg and Eggum, 2008). Vagal tone has also been correlated with compassion and openheartedness toward others from different backgrounds (Stellar et al., 2015). This perspective could also explain the soaring rates of incommunicable diseases in our modern societies, where children’s basic need for relational emotional attunement are often unmet. Having a history with chronic misattunement with one’s caregivers predisposes people to difficulties in managing challenging emotions later in life, which has implications in social life (Dozier et al., 2001). Poor affect regulation caused by early adverse experiences may manifest itself through behavioral problems in the face of stress, such as temper tantrums and emotional withdrawal (Shaver and Mikulincer, 2002).

The following section proposes that a blueprint of co-regulation between mother and baby forms during pregnancy through relational embodied processes, affecting biological systems, in which memories of early experiences are stored.

## A blueprint of emotional co-regulation

Infants’ emotions are embedded in playful relationships, which therefore shape neurobiological development (Trevarthen and Aitken, 2001). Early experiences have profound effects on multiple biological systems involving emotions, cognitions, and symbols. Our bodies (e.g., breathing and heart rhythms, muscular tensions, posture) carry the traces of our experiences, including those in prenatal life. The kinds of emotional experience that the infant has with his caregivers, for instance whether pleasurable or traumatic, are “biologically embedded.” While developing in another human body, the infant absorbs all the internal chemistry of maternal emotional/mental states (e.g., stress-hormone cortisol or feel-good hormones) through the placenta (Chamberlain, 2003; Glover, 2014). These influence gene activity and early programming of the hypothalamus-pituitary–adrenal axis of developing unborn infant (Matthews, 2002). In the prenatal and perinatal period of human life – the most critical ones – patterns of self-regulation may be forming because of shared energy and information flow stored in body memory, unborn infant brain’s extraordinary plasticity and responsiveness to maternal emotions, mind states, and communications (Sansone, 2021). A blueprint of co-regulation between mother and baby forms during pregnancy and continues to grow particularly fast during infancy in synchrony with the mammalian caregiving nature and its interactive nature (Sansone, 2021).

After nine months’ gestational synchrony, human mothers and newborns under natural conditions continue the interactional synchrony of sound and movement within the first hours after birth (Condon and Sander, 1974; Papousek and Papousek, 1992; Sansone, 2021). Repeated, positive synchronized mother-infant interactions organize, from prenatal life, the infant’s capacities for self-regulation through proper functioning of the brain leading to integration (Papousek and Papousek, 1992; Sansone, 2021). Perceived stress interferes with the flow of information, and it becomes more difficult for the caregiver to synchronize their response with flexibility, facilitating co-regulation (Sansone, 2021). The attention of a mindful mother opens the way to a prenatal attuned relationship and prepares for continued synchrony after birth (Sansone, 2021; Sansone, 2024). Sansone proposes that ideally with a mindfulness facilitator, this practice can mitigate the effects of trauma and mental challenges, which undermine that foundational synchrony. Therefore, reducing maternal distress during pregnancy and mitigating the risk of postnatal mental health disorders and their impact on infant development is a vital public health priority.

The following section advances a case for the centrality of pre and perinatal mindful parenting in the early-years development of emotion regulation, and, by extension, human flourishing, which represents a novel contribution to the developmental discourse. I propose that being a mindful mother, aware of the effects of the relational bond between herself and her baby, can help mitigate maternal distress during pregnancy and postnatal mental health disorders, reducing the risk of developmental disorders in the baby and child.

## The value of mindfulness to support emotional co-regulation

It is well established that early disruptions of the mother-infant attachment relationship may have adverse consequences on brain plasticity, integration, and resilience, and predisposes to later psychological disorders and suffering. Large and consistent body of developmental neuroscience research confirms the central role of the early attachment relationships in the neurobehavioral development, therefore future social–emotional and stress-regulation capacities of the developing individual and across generations (Cirulli et al., 2003; Brassel et al., 2020). A deeper understanding of the elements of support strategies that can mitigate the risks of postnatal depression, anxiety and stress could help maternal healthcare services provide prenatal support programs enabling mothers to cope with the challenges of the transition to childbirth and parenting (Sansone, 2024). This would minimize the risk of mental health and mother-infant relationship issues postpartum, thus facilitating emotional co-regulation and attunement between caregiver and infant leading to secure attachment, healthy social relationships, especially intimate, and human flourishing.

Mindfulness-based programs are a relatively new approach to the prevention and treatment of mental health problems. Mindfulness is a quality of human consciousness that can be independently assessed and is popularly defined by Jon Kabat Zin as “the awareness that arises from paying attention on purpose, in the present moment, and non-judgmentally, to the unfolding of experience moment by moment (Kabat-Zinn, 2003, p. 145). Mindfulness allows us to witness sensations, feelings, and thoughts as they arise in our body and mind, as ‘objects’ which can be observed directly without cognitive evaluation or elaboration, thus enabling us not to feel overwhelmed by them. Mindfulness promotes the internal attunement that is required by the interpersonal attunement between the caregiver and child that is foundation of secure attachment (Siegel, 2007).

Therefore, participating in a mindfulness-based program that significantly reduces levels of stress, depression, and anxiety and improves maternal wellbeing could reduce the risk for psychological disorders and health problems in the infant and child. Studies provided consistent evidence of mindfulness practices improving health outcomes during pregnancy and the post-partum period, promoting healthy behaviors that support the relationship between mothers and fathers and the transition to parenthood (Dhillon et al., 2017; Babbar et al., 2021; Leavitt et al., 2023). Mindfulness has been considered a protective factor fostering positive attachment and child development and behavior outcomes (Waters, 2016).

Mindful awareness involves techniques that help cope with worry by helping an individual attend to the present rather than the past and the future (Robins et al., 2012). For pregnant women and new mothers facing the challenges and stress of a significant period of transition, such skills are of particular importance to connect with their own feelings and thus to those of the baby to foster emotional co-regulation. Many parents find themselves beset by everyday preoccupations and expectations, which may generate stress and dissatisfaction with their lives. If pregnant mothers suffer from stress, depression, and/or anxiety, this has been found to impact their capacity to pick up their infant’s body cues and respond to their needs, affecting the future regulatory processes within the mother-infant relationship (Montirosso et al., 2022).

By practicing present-moment awareness of both their child and their own thoughts and emotions without judgment and accepting them for what they “are,” the parents may develop protective psychological strategies (Duncan et al., 2015). Research found that higher levels of mindfulness during pregnancy were negatively associated with depressive symptoms and positively associated with quality of prenatal attachment (Hicks et al., 2018; Sansone, 2024). These findings highlight the importance of promoting mindfulness, especially in parents at risk for depression or poor prenatal bonding. Antenatal maternal mindfulness has been associated with better self-regulation and lower levels of negative affect in 10-month-old infants (van den Heuvel et al., 2015). Studies identified associations between maternal mindfulness and response to infant stress with reduced reactivity, which indicates more responsive and attuned parenting behavior (Waters, 2016; Pickard et al., 2017). A wealth of research and theory on the implications of mindfulness for emotional experience shows that positive effects of mindfulness on emotional regulation, leading to behavioral change. A review of the literature exploring a variety of models of mindfulness revealed that mindfulness appears to improve emotion regulation by some of several mechanisms including (a) nonjudgmental awareness of challenging states which results in increased willingness to experience challenging emotions (b) a reduced reactivity to emotional stimuli and situations (c) increased emotional stability (Heppner et al., 2015).

There are associations between mindfulness awareness, secure attachment, and right hemisphere involvement outcomes. They all promote emotional regulation, wellbeing, and resilience. The interpersonal attunement that is the essence of secure attachment corresponds to the internal attunement in mindful awareness (Siegel, 2007). Both forms of attunement promote the capacity for intimate relationships, resilience, and well-being. Studies of secure attachment and those of mindful meditation have overlapping findings (Kabat-Zinn, 2003; Sroufe et al., 2005). They have found that both secure attachment and mindfulness meditation involve the growth of brain integrative areas, the corpus callosum, the hippocampus, and the prefrontal cortex and the overall connectome, which are also associated with well-being and human flourishing. In particular, the functions of the prefrontal cortex include regulation of body systems, balancing emotions, attuning to others, modulating fear, responding flexibly, empathy and compassions, intuition, and moral behavior (Siegel, 2007).

**Figure 1:**
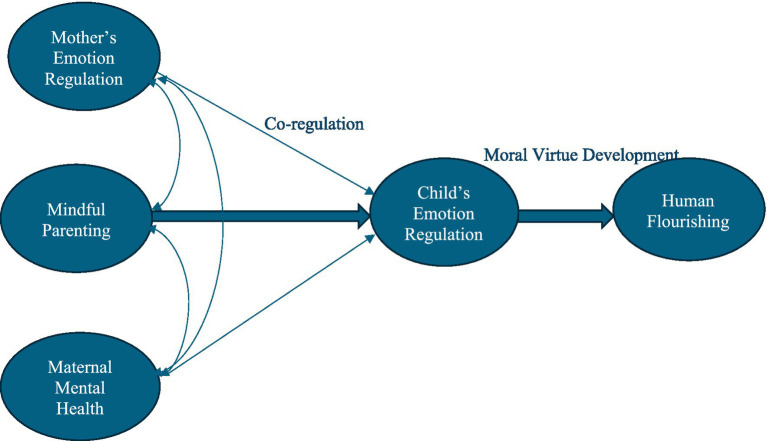
Relationship between mindful parenting, child’s emotional regulation and human flourishing, with the mediating role of maternal mental health and emotional regulation.

Figure 1 graphically represents the hypothesized relationships between mindful parenting, child’s emotional regulation and human flourishing, with the mediating role of maternal mental health and emotion regulation and mother-baby co-regulation.

Finally, the presented evidence and insights have led to the highlight of clinical implications and proposal of future directions in the following section.

## Clinical implications and future directions

Mindful awareness and all the related abilities fostered by it allows for integrative functioning of the nervous system through shared meaningful timing and gestures at the infant’s own pace and without rushing. Therefore, promoting parents’ mental wellbeing, acceptance of challenging emotions both within themselves and their infant, thus emotional regulation, and attuned responsiveness through mindfulness-based practices can impact their children’s capacity for emotional regulation, resilience, and human flourishing. In instances of maternal and infant stressors that represent a threat to their mental health and the formation of co-regulatory capacities, health enhancement strategies should be adopted by pre and perinatal healthcare providers with a focus on fostering parental mindfulness and parent-infant attuned relationship (Sansone, 2024). It has been widely recognized that this early relationship from pregnancy lays the foundation for later child development and health well into adulthood (Cirulli et al., 2003; Brassel et al., 2020).

Mindfulness practice and a mindfulness-oriented therapy promote both sensitiveness and reflective functioning fundamental for parents’ responsiveness and leading to the child’s emotional self-regulation. In particular, mindfulness-based interventions encourage awareness of emotions and body sensations, enhancing stress tolerance and reducing reactivity, all abilities improving responsiveness, understanding of an infant’s needs and feelings and emotional regulation, thus important for parenting (Hall et al., 2015).

These studies suggest that the isolation and human disconnection generated by our modern societies have impacted brain areas involved in our capacity for empathy, compassion, and all it goes with mindfulness. It is therefore insufficient to discuss human social functioning only as a psychological phenomenon. A crucial step toward developing wellbeing and human flourishing is the restoration or development of capacities for reciprocal communication from the very beginning of life. But this comes from community support and cooperation, not from isolation. Limbic resonance or relational attunement leads us toward unconditional love and reciprocity (Lewis et al., 2000). Relationships, to be attuned and emotionally regulated, especially with babies and young children, require practicing embodied emotional presence and slowing down. Mothers have evolved to be supported by a mindful community allowing them to nurture an undisturbed attuned relationship.

Practicing mindfulness and attending to others lead to the development of receptive attention and human values shifting. Healthcare providers’ positive altered traits fostered by mindfulness practice can benefit parenting and child development through a right brain to-right brain communication and heart-to-heart resonance. Neuroplasticity offers a scientific basis for a way of creating those lasting qualities of being we find in yogis and monks with repeated training. The benefits of mindfulness practice go beyond the health spectrum, including human and moral virtues of being and mindset shifting, which significantly promote parenting and attachment and a peaceful society.

## Data availability statement

The original contributions presented in the study are included in the article, further inquiries can be directed to the corresponding author.

## Author contributions

AS: Writing – original draft, Writing – review & editing.

## References

[ref1] Aristotle (1988). Nicomachean ethics WD. Ross, Trans. London, UK: Oxford University Press.

[ref2] Babbar S. Oyarzabal A. J. Oyarzabal E. A. (2021). Meditation and mindfulness in pregnancy and postpartum: a review of the evidence. Clin. Obstet. Gynecol. 64, 661–682. doi: 10.1097/GRF.0000000000000640, PMID: 34162788

[ref3] Brassel A. Townsend M. L. Pickard J. A. Grenyer B. F. S. (2020). Maternal perinatal mental health: associations with bonding, mindfulness, and self-criticism at 18 months' postpartum. Infant Ment. Health J. 41, 69–81. doi: 10.1002/imhj.21827, PMID: 31486523

[ref4] Calkins S. D. (1997). Cardiac vagal tone indices of temperamental reactivity and behavioural regulation in young children. Dev. Psychobiol. 31, 125–135. doi: 10.1002/(SICI)1098-2302(199709)31:2<125::AID-DEV5>3.0.CO;2-M, PMID: 9298638

[ref5] Chamberlain D. B. (2003). Communicating with the mind of a prenate: guidelines for parents and birth professionals. J. Prenat. Perinat. Psychol. Health. 18, 95–108.

[ref6] Cirulli F. Berri A. Alleva E. (2003). Early disruption of the mother-infant relationship: effects on brain plasticity and implications for psychopathology. Neurosci. Biobehav. Rev. 27, 73–82. doi: 10.1016/S0149-7634(03)00010-1, PMID: 12732224

[ref7] Condon W. S. Sander L. W. (1974). (1974). Neonate movement is synchronised with adult speech: interactional participation and language acquisition. Science 183, 99–101. doi: 10.1126/science.183.4120.99, PMID: 4808791

[ref8] Dhillon A. Sparkes E. Rui V. D. (2017). Mindfulness-based interventions during pregnancy: a systematic review and meta-analysis. Mindfulness 8, 1421–1437. doi: 10.1007/s12671-017-0726-x, PMID: 29201244 PMC5693962

[ref9] Dimberg U. Petterson M. (2000). Facial reactions to happy and angry facial expressions: evidence for right hemisphere dominance. Psychophysiology 37, 693–696. doi: 10.1111/1469-8986.3750693, PMID: 11037045

[ref10] DiPietro J. A. (2010). Psychological and psychophysiological considerations regarding the maternal-foetal relationship. Infant Child Dev. 19, 27–38. doi: 10.1002/icd.651, PMID: 20228872 PMC2835168

[ref11] Dozier M. Stovall K. C. Albus K. E. Bates B. (2001). Attachment for infants in foster care: the role of caregiver state of mind. Child Dev. 72, 1467–1477. doi: 10.1111/1467-8624.00360, PMID: 11699682

[ref12] Duncan L. G. Coatsworth J. D. Gayles J. Geier M. H. Geenberg M. T. (2015). Can mindful parenting be observed? Relationships between observational ratings of mother-youth interactions and mothers-self-report of mindful parenting. J. Fam. Psychol. 29, 276–282. doi: 10.1037/a0038857, PMID: 25844494 PMC4586022

[ref13] Eisenberg N. Eggum N. D. (2008). “Empathy-related and prosocial responding: conceptions and correlates during development” in Cooperation: The political psychology of effective human interaction. eds. Sullivan B. A. Snyder M. Sullivan J. L. (Blackwell Publishing), 53–74.

[ref14] Ferrari G. A. Nicolini Y. Demuru E. Tosato C. Hussain M. Scesa E.. (2016). Ultrasonographic investigation of human foetus responses to maternal communicative and non-communicative stimuli. Front. Psychol. 7:160. doi: 10.3389/fpsyg.2016.00354, PMID: 27014160 PMC4792883

[ref15] Fromm E. (1956). The heart of loving: An enquiry into the nature of love. New York: Harper & Row.

[ref16] Glover V. (2014). Maternal depression, anxiety and stress during pregnancy and child outcome; what needs to be done. Best Pract. Res. Clin. Obstet. Gynaecol. 28, 25–35. doi: 10.1016/j.bpobgyn.2013.08.017 24090740

[ref17] Greenspan S. I. Shanker S. I. (2004). The first idea. Cambridge, MA: Da Capo Press.

[ref18] Groves D. A. Brown V. J. (2005). Vagal nerve stimulation: a review of its applications and potential mechanisms that mediate its clinical effects. Neurosci. Biobehav. Rev. 29, 493–500. doi: 10.1016/j.neubiorev.2005.01.004, PMID: 15820552

[ref19] Hall H. G. Beattie J. Lau R. East C. Anne B. M. (2015). Mindfulness and perinatal mental health: a systematic review. Women. Birth 29, 62–71. doi: 10.1016/j.wombi.2015.08.006, PMID: 26346905

[ref20] Hayes L. J. Goodman S. H. Carlson E. (2013). Maternal antenatal depression, and infant disorganized attachment at 12 months. Attach Hum. Dev. 15, 133–153. doi: 10.1080/14616734.2013.743256, PMID: 23216358 PMC3594350

[ref21] Heppner W. L. Spears C. A. Vidrine J. I. Wetter D. W. (2015). “Mindfulness and emotion regulation” in Handbook of mindfulness and self-regulation. eds. Ostafin B. Robinson M. Meier B. (New York, NY: Springer).

[ref22] Hicks L. M. Dayton C. J. Brown S. Muzik M. Raveau H. (2018). Mindfulness moderates depression and quality of prenatal attachment in expectant parents. Mindfulness 9, 1604–1614. doi: 10.1007/s12671-018-0907-2

[ref23] Hrdy S. B. (2009). Mothers and others: The evolutionary origins of mutual understanding. Cambridge, MA: Belknap Press.

[ref24] Johnson M. (1993). Moral imagination: Implications of cognitive science for ethics. Chicago: University of Chicago Press.

[ref25] Kabat-Zinn J. (2003). Mindfulness-based interventions in context: past, present, and future. Clin. Psychol. Sci. Pr. 10, 144–156.

[ref26] Kok B. E. Fredrickson B. L. (2010). Indexed by vagal tone, reciprocally and prospectively predicts positive emotions and social connectedness. Biol. Psychol. 85, 432–436. doi: 10.1016/j.biopsycho.2010.09.005, PMID: 20851735 PMC3122270

[ref27] Konner M. (2005). “Hunter-gatherer infancy and childhood. The! Kung and others” in Hunter-gatherer childhood: Evolutionary, developmental and cultural perspectives. eds. Hewlett B. Lamb M. (New Brunswick, NJ: Aldine/Transaction).

[ref28] Kristjánsson K. (2019). Flourishing as the aim of education: A neo-Aristotelian view. London, UK: Routledge.

[ref29] Kristjánsson K. Fowers B. (2023). 2023 Phronesis: Retrieving practical wisdom in psychology, philosophy, and education. Oxford: Oxford University Press.

[ref30] Leavitt C. E. Wikle J. S. Kramer Holmes E. Pierce H. Eyring J. Gibby A. L.. (2023). Mindfulness and individual, relational, and parental outcomes during the transition to parenthood. J. Soc. Pers. Relat. 40, 1422–1447. doi: 10.1177/02654075221137870

[ref31] Lewis M. D. (2005). Bridging emotions theory and neurobiology through dynamic systems modelling. Behav. Brain Sci. 28, 169–194. doi: 10.1017/S0140525X0500004X, PMID: 16201458

[ref32] Lewis T. Amini F. Lannon R. (2000). A general theory of love. New York: Vintage.

[ref33] MacMurray J. (1992). Reason and emotion. Amherst, NY: Humanity Books.

[ref34] Mason Z. S. Briggs R. D. Silver E. J. (2011). Maternal attachment feelings mediate between maternal reports of depression, infant social-emotional development, and parenting stress. J. Reprod. Infant Psychol. 29, 382–394. doi: 10.1080/02646838.2011.629994

[ref35] Matthews S. G. (2002). Early programming of the hypothalamo-pituitary-adrenal axis. Trends Endocrinol. Metab. 13, 373–380. doi: 10.1016/s1043-2760(02)00690-2 12367818

[ref36] McGowan P. O. Sasaki A. Huang T. C. Unterberger A. Suderman M. Ernst C.. (2008). Promoter-wide hypermethylation of the ribosomal RNA gene promoter in the suicide brain. PLoS One 3:e2085. doi: 10.1371/journal.pone.0002085, PMID: 18461137 PMC2330072

[ref37] Montirosso R. Mascheroni E. Mariani Wigley I. L. C. (2022). “Maternal embodied sensitivity: could interoception support the mother’s ability to understand her infant’s signals?” in Key topics in perinatal mental health. eds. Percudani M. Bramante A. Brenna V. Pariante C. (Switzerland AG: Springer Nature).

[ref38] Narvaez D. (2014). Neurobiology and the development of human morality. Evolution, culture, and wisdom. New York: Norton.

[ref39] Panksepp J. (1998). Affective neuroscience: The foundations of human and animal emotions. New York: Oxford University Press.

[ref40] Papousek H. Papousek M. (1992). Beyond emotional bonding: the role of preverbal communication in mental growth and health. Infant Ment. Health J. 13, 43–53. doi: 10.1002/1097-0355(199221)13:1<43::AID-IMHJ2280130108>3.0.CO;2-R

[ref41] Pickard J. A. Townsend M. L. Caputi P. Grenyer B. F. (2017). Top-down and bottom-up: the role of social information processing and mindfulness as predictors in maternal-infant interaction. Infant Ment. Health J. 39, 44–54. doi: 10.1002/imhj.21687, PMID: 29266312

[ref42] Porges S. W. (2011). The polyvagal theory. Neurophysiological foundations of emotions attachment, communication, self-regulation. Norton: W. W. Norton & Co.

[ref43] Porges S. W. Doussard-Roosevelt J. A. Maiti A. K. (1994). Vagal tone and the physiological regulation of emotion. Monogr. Soc. Res. Child Dev. 59, 167–186. doi: 10.1111/j.1540-5834.1994.tb01283.x 7984159

[ref44] Porter C. L. (2003). Coregulation in mother-infant dyads: links to infants' cardiac vagal tone. Psychol. Rep. 92, 307–319. doi: 10.2466/pr0.2003.92.1.307, PMID: 12674298

[ref45] Robins C. J. Keng S. L. Ekblad A. G. Brantley J. G. (2012). Effects of mindfulness-based stress reduction on emotional experience and expression: a randomised controlled trial. J. Clin. Psychol. 68, 117–131. doi: 10.1002/jclp.20857 22144347

[ref46] Sansone A. (2021). Cultivating mindfulness to raise children who thrive: Why human connection from before birth matters. London, UK: Routledge.

[ref47] Sansone A. (2024). Participation in an online prenatal mindfulness-relationship-based (PMRB) program: outcomes for maternal mindfulness, mental health, interoception, and mother-infant relationship during pregnancy and post-partum. OBM Integr. Complem. Med. 9, 1–43. doi: 10.21926/obm.icm.2401001

[ref48] Schore A. N. (1994). Affect regulation and the origin of the self: The neurobiology of emotional development. Mahwah, NJ: Erlbaum.

[ref49] Schore A. N. (2005). Back to basics: attachment, affect regulation, and the developing right brain: linking developmental neuroscience to pediatrics. Pediatr. Rev. 26, 204–217. doi: 10.1542/pir.26.6.204 15930328

[ref50] Schore A. N. (2021). The interpersonal neurobiology of intersubjectivity. Front. Psychol. 12:648616. doi: 10.3389/fpsyg.2021.648616, PMID: 33959077 PMC8093784

[ref51] Shaver P. R. Mikulincer M. (2002). Attachment-related psychodynamics. Attach Hum. Dev. 4, 133–161. doi: 10.1080/14616730210154171, PMID: 12467506

[ref52] Siegel D. J. (2007). The mindful brain. Reflection and attunement in the cultivation of well-being. New York: Norton. 10.1176/appi.ajp.2007.07081292 22688157

[ref53] Siegel D. J. (2023). IntraConnected. New (me+we). As the integration of self, identity, and belonging. Norton: W. W. Norton & Co.

[ref54] Sroufe L. A. Egeland B. Carlson E. A. Collins W. A. (2005). The development of the person: The Minnesota study of risk and adaptation from birth to adulthood. New York: Guilford Press.

[ref55] Stellar J. E. Cohen A. Oveis C. Keltner D. (2015). Affective and physiological responses to the suffering of others: compassion and vagal activity. J. Pers. Soc. Psychol. 108, 572–585. doi: 10.1037/pspi0000010 25621856

[ref56] Trevarthen C. Aitken K. J. (2001). Infant intersubjectivity; research, theory, and clinical applications. J. Child Psychol. Psychiatry Allied Discip. 42, 3–48. doi: 10.1111/1469-7610.00701, PMID: 11205623

[ref57] van den Heuvel M. I. Johannes M. A. Henrichs J. Van den Bergh B. R. H. (2015). Maternal mindfulness during pregnancy and infant socio-emotional development and temperament: the mediating role of maternal anxiety. Early Hum. Dev. 91, 103–108. doi: 10.1016/j.earlhumdev.2014.12.003, PMID: 25577496

[ref9001] Verny T. R. (2021). The embodied mind: understanding the mysteries of cellular memory, consciousness, and our bodies. New York: Pegasus Books.

[ref58] Waters L. (2016). The relationship between child stress, child mindfulness and parent mindfulness. Psychology 7, 40–51. doi: 10.4236/psych.2016.71006

[ref59] Weller A. Feldman R. (2003). Emotion regulation and touch in infants: the role of cholecystokinin and opioids. Peptides 24, 779–788. doi: 10.1016/S0196-9781(03)00118-9 12895666

[ref60] Zimmer-Gembeck M. J. Rudolph J. Kerin J. Bohadana-Brown G. (2022). Parent emotional regulation: a meta-analytic review of its association with parenting and child adjustment. Int. J. Behav. Dev. 46, 63–82. doi: 10.1177/01650254211051086

